# Association between electronic nicotine delivery systems and electronic non-nicotine delivery systems with initiation of tobacco use in individuals aged < 20 years. A systematic review and meta-analysis

**DOI:** 10.1371/journal.pone.0256044

**Published:** 2021-09-08

**Authors:** Sze Lin Yoong, Alix Hall, Heidi Turon, Emily Stockings, Alecia Leonard, Alice Grady, Flora Tzelepis, John Wiggers, Hebe Gouda, Ranti Fayokun, Alison Commar, Vinayak M. Prasad, Luke Wolfenden

**Affiliations:** 1 School of Health Sciences, Swinburne University of Technology, Hawthorn, Victoria, Australia; 2 School of Medicine and Public Health, University of Newcastle, Callaghan, NSW, Australia; 3 Hunter Medical Research Institute, New Lambton Heights, NSW, Australia; 4 Priority Research Centre for Heath Behaviour, University of Newcastle, Callaghan, NSW, Australia; 5 Hunter New England Population Health, Wallsend, NSW, Australia; 6 National Drug and Alcohol Research Centre, University of New South Wales, Randwick, NSW, Australia; 7 No Tobacco Unit, Department of Health Promotion, World Health Organization, Geneva, Switzerland; University of California San Francisco (retired), UNITED STATES

## Abstract

**Background:**

This systematic review described the association between electronic nicotine delivery systems and electronic non-nicotine delivery systems (ENDS/ENNDS) use among non-smoking children and adolescents aged <20 years with subsequent tobacco use.

**Methods:**

We searched five electronic databases and the grey literature up to end of September 2020. Prospective longitudinal studies that described the association between ENDS/ENNDS use, and subsequent tobacco use in those aged < 20 years who were non-smokers at baseline were included. The Joanna Briggs Institute Critical Appraisal Checklist was used to assess risk of bias. Data were extracted by two reviewers and pooled using a random-effects meta-analysis. We generated unadjusted and adjusted risk ratios (ARRs) describing associations between ENDS/ENNDS and tobacco use.

**Findings:**

A total of 36 publications met the eligibility criteria, of which 25 were included in the systematic review (23 in the meta-analysis) after exclusion of overlapping studies. Sixteen studies had high to moderate risk of bias. Ever users of ENDS/ENNDS had over three times the risk of ever cigarette use (ARR 3·01 (95% CI: 2·37, 3·82; p<0·001, I^2^: 82·3%), and current cigarette use had over two times the risk (ARR 2·56 (95% CI: 1·61, 4·07; p<0·001, I^2^: 77·3%) at follow up. Among current ENDS/ENNDS users, there was a significant association with ever (ARR 2·63 (95% CI: 1·94, 3·57; p<0·001, I^2^: 21·2%)), but not current cigarette use (ARR 1·88 (95% CI: 0·34, 10·30; p = 0·47, I^2^: 0%)) at follow up. For other tobacco use, ARR ranged between 1·55 (95% CI 1·07, 2·23) and 8·32 (95% CI: 1·20, 57·04) for waterpipe and pipes, respectively. Additionally, two studies examined the use of ENNDS (non-nicotine devices) and found a pooled adjusted RR of 2·56 (95% CI: 0·47, 13·94, p = 0.035).

**Conclusion:**

There is an urgent need for policies that regulate the availability, accessibility, and marketing of ENDS/ENNDS to children and adolescents. Governments should also consider adopting policies to prevent ENDS/ENNDS uptake and use in children and adolescents, up to and including a ban for this group.

## Introduction

Electronic Nicotine Delivery Systems (ENDS) and Electronic Non-Nicotine Delivery Systems (ENNDS) are systems that use devices to heat liquids to create aerosols that are inhaled by users. These are most commonly in the form of an ‘e-cigarette’, but come in other forms (e.g ‘e-pipe’, ‘e-shisha’, ‘e-cigars’). [1] These systems typically contain flavourings, propylene glycol, glycerine and, for ENDS–nicotine. ENDS/ENNDS were first introduced into markets in the 2000s and have been promoted aggressively by manufacturers as “reduced harm products” or “alternatives” to conventional cigarettes. [2] The use of ENDS/ENNDS among children and adolescents however is increasing in some countries, especially among those who had never used tobacco, [3] indicating that such products are not solely used or targeted at adults. [4] In many developed countries, including Canada and the United States (US), ENDS/ENNDS use far surpasses the rates of tobacco use among adolescents in high school. [5–7]

Of concern is an increasing body of evidence suggesting ENDS/ENNDS use may accrue a range of health risks for different age groups. [8, 9] Constituents of e-liquids, such as propylene glycol and glycerine form toxic aldehydes when heated, of which the long-term effects of exposure remains unknown. [10] ENDS/ENNDS use can also impact on the respiratory system and is associated with adverse effects on the developing brain. [10] A recent position statement by the European Association of Preventive Cardiology reported that e-cigarettes may have negative effects on cardiovascular health for both adolescents and adults. [11] There is a rapidly developing empirical evidence describing a longitudinal association between ENDS/ENNDS and cigarette use among young people.

The first review of three prospective cohort studies in those <20 years in 2016 commissioned by the World Health Organization (WHO), reported that non-smoking e-cigarette users had twice the odds of being a conventional cigarette user at follow-up. [3] Since then, there have been several systematic reviews including at time of conducting our review, the most recent by Khouja and colleagues. [12–16] The review by Khouja included 17 studies with individuals aged <30 years, published up to November 2018. The majority of studies were conducted in the US and found a significant adjusted association between ENDS/ENNDS use among non-smokers at baseline and later cigarette use (OR: 2·92 (95% CI 2·30, 3·71). Since this review a number of longitudinal studies have been published from a broader range of countries. [17, 18] An updated systematic review to reflect the contemporary evidence is warranted, as more countries are enacting or planning to enact policy or programs to deter ENDS/ENNDS use in young people globally. [19]

Therefore, this review synthesised findings from studies assessing the longitudinal association between ENDS and/or ENNDS use and later cigarette (primary outcome) and other tobacco product initiation (secondary outcome) among children and adolescents aged < 20 years, who were never smokers at baseline. Additionally, it sought to describe the longitudinal association of ENNDS and flavoured ENDS/ENNDS and subsequent tobacco use.

## Methods

### Search strategy and selection criteria

This systematic review and meta-analysis is undertaken consistent with guidance by Joanna Briggs Institute (JBI) [20] and reported in accordance with Meta-analysis of Observational Studies in Epidemiology (MOOSE) guidelines. [21] It was prospectively registered in the PROSPERO database (CRD42020199485).

Studies were included if they were prospective longitudinal studies assessing the relationship between ENDS and/or ENNDS use at baseline and initiation of cigarette and other tobacco products at follow-up, among children and adolescents aged less than 20 years who were non-tobacco users at baseline. Case control, cross-sectional and retrospective studies were excluded to capture only studies with the lowest risk of bias for assessing association. [22] There were no restrictions on the year of publication, length or location of the study, peer review status, or language of publication.

We conducted an electronic search of the following databases: Medline, Web of Science, CINAHL, Embase and Wiley Cochrane Library using search terms for the following *‘electronic nicotine delivery systems (ENDS) electronic non-nicotine delivery systems (ENNDS)*,*’* AND *‘prospective studies’* AND *‘children and adolescents’* (see S1 Appendix for search strategy) on the September 2020. The reference lists of all relevant reviews and eligible papers were also screened. We undertook a grey literature search based on guidance from previous reviews, [23] which included searching OpenGrey (a grey literature database) and Google and Google scholar to identify relevant studies using the following terms ‘*electronic cigarette’*, ‘*e-cigarette’*, ‘*electronic nicotine delivery systems (ENDS)*, *‘electronic non-nicotine delivery systems (ENNDS)’*, *‘e-hookah’ and ‘juul’*. The first 500 titles of each search were sorted by relevance were assessed by one reviewer in October 2020 (SLY).

An information specialist used EndNote version X9.2 software (Thomson Reuters, PA, U.S.) to filter duplicate studies. Title and abstract screening were undertaken using Covidence software [24] by two reviewers, and discrepancies resolved by consensus (SLY, AH). Full text was obtained and assessed for eligibility in accordance with the criteria described above by two reviewers (AL, ES). All conflicts were resolved by discussion and included a third reviewer (SLY), where necessary.

All data were extracted by a first reviewer (AG, FT, SLY or HT) and double checked by a second reviewer not involved in the original extraction of the study (SLY, HT or AL). Discrepancies were highlighted and checked by a third reviewer (AH). The following information was extracted: participant characteristics, study design, country, data collection modality and measure, sampling frame and recruitment, proportion and number of ENDS/ENNDS users separately where reported, tobacco users as well as non-users at each time point, relevant measures of association between ENDS and ENNDS users and future cigarette and other tobacco product initiation (e.g. risk ratios, odds ratios), estimates of variance and covariates adjusted for, follow-up time points, type of tobacco products assessed and flavours.

The JBI Critical Appraisal Checklist for prevalence studies was used to assess the quality of each study by two reviewers (AG, HT, FT, AL). [25] Discrepancies were checked by a third reviewer (SLY). The tool consists of nine items examining the following: sample representativeness, sampling methods, adequacy of sample size, participant and setting descriptions, coverage of sample, objectivity and reliability of measures, appropriateness of statistical analysis, confounding factors identified and accounted for, and objective classification of subpopulations (Yes; No; Unclear; and N/A). An additional tenth criterion relating to participant retention was added to allow for assessment of attrition bias. Two reviewers also assessed four supplementary criteria detailed in the Bradford-Hill criteria relevant to establishing causality between exposure and outcome. [26] (see S1 Table)

### Data analysis

All analyses were undertaken using Stata version 14.2. [27] Effect estimates (extracted or converted to Risk Ratios (RRs)) of the association between ENDS/ENNDS use at baseline and initiation of cigarette or other tobacco use at follow up were combined using the DerSimonian and Laird random effects method. [28]

The primary outcome variable was ever and current cigarette smoking. For ever cigarette smoking, this included lifetime ever use. For current cigarette use, this included use in the past 30 days, frequent and daily cigarette use. The exposure variable was ever and current ENDS/ENNDS use. For ever use of ENDS and/or ENNDS, this was defined as lifetime ever use. For current use of ENDS and/or ENNDS, this included use in the past 30 days, recent use and self-defined current use.

A p-value of 0·05 was used to determine a statistically significant association. Where it was not appropriate to undertake a meta-analysis (due to heterogeneity or small number of studies), study findings were narratively described.

For studies that did not report the unadjusted RRs, these were calculated using the data extracted from the original study or converted from an odds ratio (OR). In instances where studies reported an adjusted odds ratio (AOR) rather than an adjusted RR, these were also converted to an RR. The formula from the Cochrane Handbook (Section 15.4.4.4) [29] was used to convert ORs to RRs. The ACR was calculated on a per study basis as the risk of later smoking among controls, whereby the control was defined as no ENDS/ENNDS use at baseline. In instances where a study did not provide sufficient data to calculate a study-specific ACR, the average ACR from other studies was used.

Where multiple follow-up points were available, the furthest time from baseline was included. Additionally, when a study reported a slight variation for the same outcome, using overlapping datasets, the outcome most closely aligned with the aims was chosen. Where multiple effect estimates exist controlling for different confounders, we included the ones that controlled for demographics that had evidence of association with tobacco uptake (sex, age, socioeconomic status and susceptibility to tobacco use), where available.

A number of planned subgroup analyses were undertaken [12] including: country (grouped into US, United Kingdom (UK) and other), study quality (<7 and 7 or more on the Joanna Briggs scale), and Bradford-Hill’s causal inference score (> = 3 and <3). Additionally, we also undertook a subgroup analysis by length of follow up (≤ 12 months and >12 months) and publication year (≤ 2018 and >2018). We planned to undertake sensitivity analysis by funding source (e.g. industry/non-industry), however no industry funded studies were included in the meta-analysis.

Heterogeneity of study effect estimates were evaluated using the I^2^ statistic. A funnel plot and the Duval and Tweedie trim-and-fill method was used to examine possible publication bias and provide an estimate of the bias-adjusted pooled estimates. [30–32]

## Results

**Fig 1:**
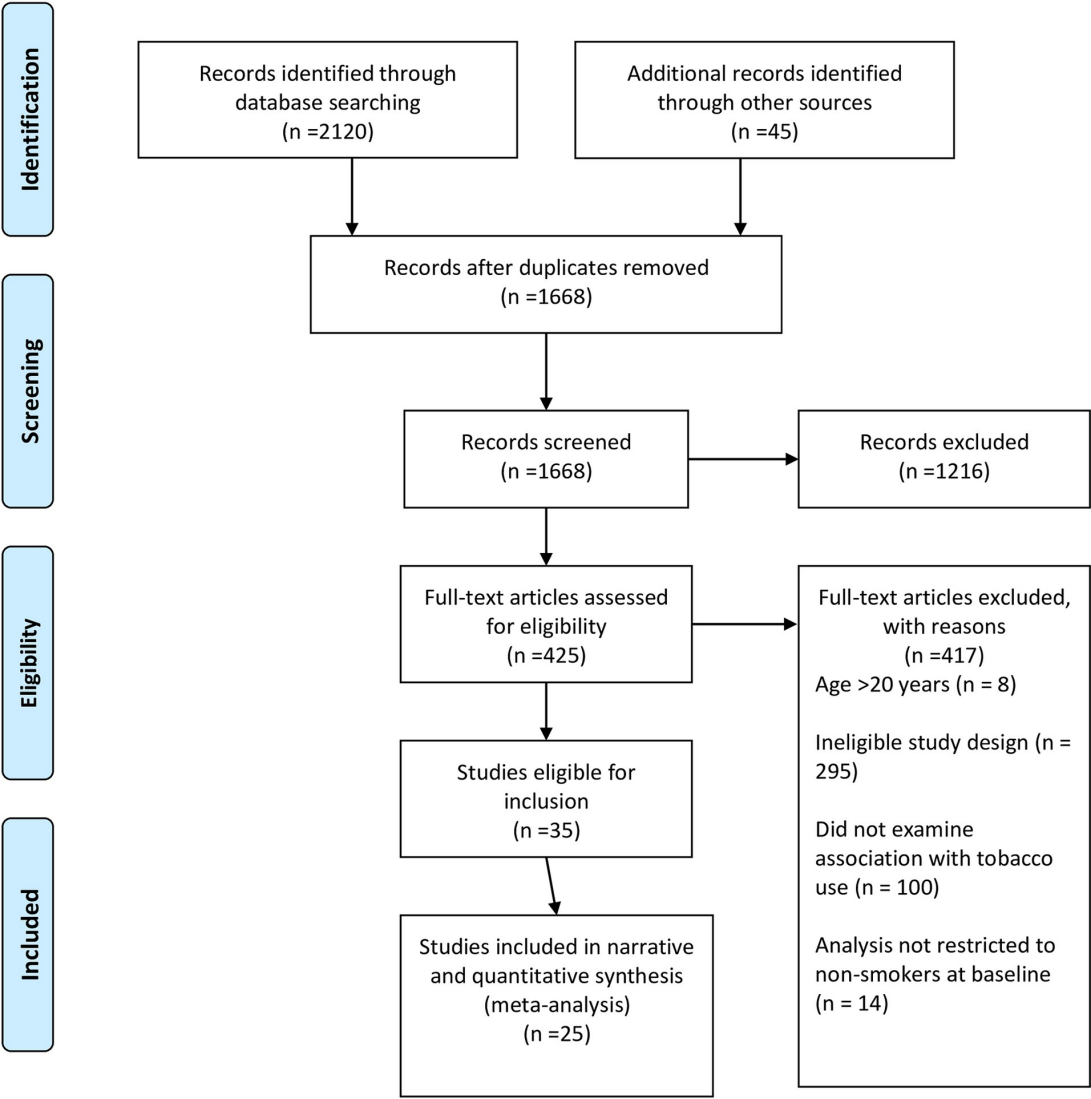
PRISMA flowchart outlining study inclusion and exclusion.

Of the 1,668 studies included after removal of duplicates, 452 articles underwent full text screen, of which 35 were included (see Fig 1). Of those, 10 were excluded from the final analysis as there was overlap of data with other studies included in this review. A total of 25 studies were included in the review, of which 23 were included in any meta-analyses (Fig 1). [17, 18, 33–53]

The studies were conducted in the US (n = 13), Germany (n = 3), UK (n = 2), Scotland (n = 1), Canada (n = 1), Finland (n = 1), Mexico (n = 1), Taiwan (n = 1), Netherlands (n = 1) and Romania (n = 1) with data collection occurring from 2013–2016 at baseline (see Table 1). Sample sizes ranged from 164 to 17,318 and participants were aged between 11 to 26 years (as studies were eligible for inclusion if they had a mean age of <20). The follow-up period was between six to 24 months, and all studies used self-reported measures to assess cigarette (and/or tobacco) use at follow up. Overall, 21 studies assessed cigarette smoking only as an outcome, [17, 18, 34–41, 43–47, 49–54] three assessed cigarettes and other tobacco [33, 42, 48] and one assessed other tobacco only. [55] All studies referred to ENDS/ENNDS as e-cigarettes. Two studies specifically assessed the use of non-nicotine e-cigarettes [40, 51] while one study compared flavoured and non-flavoured e-cigarettes. [54]

**Table 1:** Characteristics of study included in the review.

Author name, year of publication, geographic region	Survey name	Study design, number of time points, length of follow up	n analysed, % retention rate	Sample characteristics (at baseline) (sex, age, ethnicity)	Sampling procedure	Data collection modality	Type of ENDS assessed (specify nicotine/non-nicotine)	Main outcomes assessed (e.g. association between ever and current ENDS/ENNDS use)	Adjustments accounted for in analysis
Barrington-Trimis 2016, Southern California United States [33]	Southern California Children’s Health Study (CHS),	Longitudinal prospective cohort study, 2 time points, median follow-up length: 15·6 (IQR:12·6–18·2) months	n analysed: 149 (e-cigarette users) retention rate: 70.0%. n analysed: 154 (never e-cigarette users), retention rate: 72·3%	Male: 58·4%, Female: 41·6%, Age: Median: 17·4 (IQR:16·8–17·9) years, Race: Hispanic white: 146 (49%), Non-Hispanic white: 126 (42·3%), Other: 26 (8·7%) Education (highest parental): ≤12th grade: 86 (30·4%), Some college: 100 (35·3), College degree of higher: 97 (34·3%)	Exposure frequency-matched cohort study design. Never-smoking e-cigarette users were contacted and a sample of never-smoking never e-cigarette users to complete a follow-up questionnaire.	Pen and paper	e-cigarette	Ever use of ENDS/ENNDS at baseline and ever use of tobacco AND other tobacco products (pipes, cigars, hookah, any combustible product)	Gender, ethnicity, grade and highest parental education.
Barrington-Trimis 2018 California and Connecticut United States [34]	Southern California Children’s Health Study (CHS), Happiness and Health (H&H) Study, Yale Adolescent Survey Study (YASS)	Longitudinal, prospective cohort study, 2 time points, length of follow-up: 6–12 months	CHS: Nn = 1553; response rate = 74·0%. H&H: n = 3190; response rate = 93 ·9%. YASS: N = 1404; match rate = 60·0%)	Male: 46·5%, Female: 53·5%, 9th-12 grade, Race/Ethnicity (CHS/HH/YASS) *Non-Hispanic white* n = 592 (38·1)/ n = 512 (16·0)/ n = 1198 (85·3) *Hispanic white* n = 758 (48·8) /n = 1505 (47·2) /n = 66 (4·7) *Other* n = 203 (13·1)/ n = 1173 (36·8)/ n = 140 (10·0)	The Southern California Children’s Health Study (CHS) is a population-based study of youth in 12 communities across Southern California. The Happiness and Health (H&H) Study is a population-based study of adolescents in 10 schools in the greater Los Angeles area. The Yale Adolescent Survey Study (YASS) is a cohort study. An initial sample of students was recruited from 3 high schools.	Pen and paper	e-cigarette	Ever use of e- cigarette (baseline) and ever use of cig (follow-up)	Sex, race and/or ethnicity, grade, and study.
Berry 2019 United States [35]	Population Assessment of Tobacco and Health Study (PATH)	Prospective cohort study, 2 time points, follow-up: 12–24 months	n analysed: 6123, retention rate: 80·9%	Age: 13·4 (1·2), Male: 50·5%, Female: 49·5% Ethnicity: 54·1% non-Hispanic, white, 13·9% non-Hispanic, black, 22·8% Hispanic, 9·2% non-Hispanic other	This longitudinal survey’s cohort was selected via a 4-stage, stratified probability sample that was nationally representative.	Audio computer-assisted self-interviewing.	e-cigarette	Ever use of e- cigarette (baseline) and ever use of cigarette (follow-up), ever use of e- cigarette (baseline) and current use of cigarette (follow-up).	Sex, age, race and ethnicity, parental education, urban or rural residence, living with a tobacco user, noticing tobacco warnings, tobacco advertisement receptivity, ever alcohol use, ever marijuana use, prescription drug abuse, enjoying frightening things, liking new and exciting experiences, preferring unpredictable friends, willingness to smoke in next year, curiosity about cigarettes, and susceptibility to cigarette peer pressure from friends.
Best 2017 Scotland [36]	Determining the Impact of Smoking Point-of-Sale Legislation Among Youth (DISPLAY) study	Longitudinal prospective cohort study, 2 time points, follow-up length: 12 months	n analysed: 2,680, retention rate: 70·4%	Age: 14·4 (1·58), Male/Female % NR, ethnicity NR	Schools were purposively selected to reflect two levels of urbanisation and two levels of socio-economic deprivation (derived from the population-weighted mean Scottish Index of Multiple Deprivation (SIMD) score for all data zones falling within the school catchment areas and the proportion of children from each school receiving free school meals).	Pen and paper	e-cigarette	Ever use of use-cigarette at baseline and ever use of tobacco at follow-up	Sex, age, family affluence, ethnic group, school, smoking within the family, smoking by friends and susceptibility to smoking
Chien 2019 Taiwan [17]	Taiwan Adolescent to Adult Longitudinal Study (TAALS)	Longitudinal prospective cohort study, 2 time points, follow-up: 24 months	n analysed: 12,954, retention rate: 87%	Male/Female % NR, 7th grade (n = 6667) mean age: 13 years, senior high school - 10th grade (n = 4689) mean age: 16 years), and vocational high school - 10th grade, (n = 6708) mean age: 16 years).	School was the primary sampling unit and the first wave included first-year students from junior high school, 7th grade, senior high school, 10th grade, and vocational high school students	NR	e-cigarette	Ever use of e-cigarette use at baseline and ever use of tobacco at follow-up	Smoking susceptibility at baseline, socio-demographic profile, psychological status, and peer support
Conner 2018 England [37]	NR	longitudinal prospective cohort study, 2 time points, follow-up: 12 months	n analysed: 1,726, retention rate: 56%	Male: 48%, Female: 53%, Age at BL: 13–14 years, Ethnicity NR	Data collected as part of a 4-year cluster randomised controlled trial from adolescents in 20 control schools. Adolescents matched across time points using a personally generated code.	Online	e-cigarette/ vapourisers	Ever use of e-cigarette use at baseline and ever use of tobacco at follow-up	Friend smoking, sex, family smoking, intentions, attitudes, norms, perceived behavioural control, self-efficacy, free school meals
East 2018 Great Britain [38]	2016 Action on Smoking and Health Great Britain Youth longitudinal survey	Longitudinal prospective cohort study, 2 time points, follow-up: 4–6 months	n analysed: 923, retention rate: 50%	Male: 46·4%, Female: 53·6%, Age: 38·0% 11–13 year olds, 29·3% 14–15 year olds, 32·6% 16–18 year olds, ethnicity NR	A non-probability quota sampling approach was adopted using Ipsos MORI’s online panels to recruit respondents aged 11–18 years. Quotas were set in respect of age, gender, and Government Office Region (GOR) using data from Eurostat 2012 to ensure sample representativeness.	Online	e-cigarette	Ever use of e-cigarettes use at baseline and ever use of tobacco at follow-up	Age, gender, school performance, problem behaviour, monthly alcohol use, smoking susceptibility, friend smoking, friend e-cigarettes use, parent smoking, parent e-cigarettes use, sibling smoking, sibling e-cigarettes use, public approve of smoking, public approve of e-cigarettes s
Friedman 2020 United States [54]	Population Assessment of Tobacco and Health Study (PATH)	Prospective cohort study, 2 time points, follow-up: 5 years total, including waves 1–4	n analysed: 164, Wave 1 response rates were 75% for the youth. Wave 3 response rates (within the wave 1 cohort) were 78%	Male: 51·4%, Female: 48·6%, Age: 12–17 years, no mean reported, 66·9% white	This longitudinal survey’s cohort was selected via a multistage, stratified probability sample, such that weighted analyses were nationally representative for the noninstitutionalized US civilian population	Responses were collected with audio computer-assisted self-interviewing in English or Spanish.	e-cigarettes	initiated flavoured/unflavoured current e-cigarettes use (wave 2) and cig current use (in past 30 days) wave 3	Sex, race (black and other, with white as the reference group), Hispanic ethnicity, age group, household income categories (wave 2 parental reports for youths, and an indicator for having ever tried conventional cigarettes at wave 1 as well as a missing-observation indicator for each of these variables. Additionally, youth regressions controlled for parental education at baseline (high school graduate or equivalent, some college, and college graduate, with high school graduate as the reference group).
Hammond 2017 Ontario and Alberta Canada [39]	COMPASS	Longitudinal prospective cohort study, 2 time points, follow up: 12 months	n analysed: 17,318, retention rate: 43%	Male: 46·6%, Female: 53·4%, Age: <14 - >18 years, Race/ethnicity: White: n = 14 940 (77·7), Black: n = 603 (3·1), Asian: n = 979 (5·1), Aboriginal: n = 478 (2·5), Latin American/Hispanic: n = 305 (1·6), Other/mixed: n = 1929 (10·0) Spending money, $0: n = 3605 (18·7), $1–20: n = 594 (34·1), $21–100: n = 4650 (24·1), >$100: n = 1850 (9·6),	Purposefully sampled	Pen and paper	e-cigarette	Current e-cigarette use at baseline and ever tobacco use at follow-up	Student clustering within schools (school as a random effect) and the past-wave covariables (i.e., baseline values) of age, sex, race/ethnicity, spending money and past 30-day e-cigarette use as fixed effects.
Hansen 2020a, Baden-Württemberg Mecklenburg-Western Pomerania, North Rhine-Westphalia, Rhineland-Palatinate, Schleswig-Holstein and Saxony Germany [18]	DAK prevention radar	longitudinal cohort study, 2 time points, follow-up: 24 months	n analysed: 2,388, retention rate: 56·6%	Male: 50·3%, Female: 49·6%, mean age at baseline: 12 years, type of school (grammar): 45%; Migration background: 85.1%; SES mean (SD): 6·7 (1·4)	Each state was randomly selected from one of the six Nielsen regions. A total of 627 secondary schools were identified in randomly selected sub-regions within each state, and all of them were invited to participate in the study	Mixed (online or pen and paper)	e-cigarette	Ever use of e-cigarette use at baseline and ever use of tobacco at follow-up	Age, gender, migration background, sensation seeking, school performance, alcohol consumption, SES, type of school
Hansen 2020b, Baden-Württemberg Mecklenburg-West-Pomerania, North-Rhine-Westphalia, Rhineland-Palatinate, Saxony, and Schleswig-Holstein Germany [55]	DAK prevention radar	longitudinal cohort study, 2 time points, follow-up: 12 months	n analysed: 3771, retention rate: 76·2%	Male: 51%, Female: 49%, mean age at baseline: 13·1 years, type of school (gymnasium): 51·3%	Each state was randomly selected from one of the six Nielsen regions. A total of 627 secondary schools were identified in randomly selected sub-regions within each state, and all of them were invited to participate in the study.	Mixed (online or pen and paper)	e-cigarette	Ever use of e-cigarette at baseline and ever use of hookah at follow-up	Age, gender, migration background, sensation seeking, SES, type of school, peer substance use
Kinnunen 2019 Helsinki Finland [40]	Metropolitan Longitudinal Finland (MetLoFIN)	Longitudinal cohort study, 2 time points, follow-up: 24 months	n analysed: 2, 016, retention rate: 44·9%	Male: 48·2%, Female: 51·8%, Age at baseline:15–16 years	NR	Online	Electronic Non-nicotine Delivery Systems (ENNDS)	Ever ENNDS use at baseline and current tobacco use at follow-up	Gender, SES, other tobacco product and drug use. School clustering was accounted for.
Kong 2019 California and Connecticut United States [41]	Southern California Children’s Health Study (CHS), Happiness and Health (H&H) Study, Yale Adolescent Survey Study (YASS)	Longitudinal prospective cohort study, 2 timepoints, follow-up: 12–18 months	n analysed: 4876 Retention rate: NR	Male: 46·3%, Female: 53·7%, Mean age: 15·5 years old (SD = 1·4), Ethnicity: Non-Hispanic White: 1897 (38·9), Hispanic: 1794 (36·8), Other: 1185 (24·3), Non-Hispanic Black: 143 (2·9), Asian: 566 (11·6), Other including Bi- and Multi-Racial: 476 (9·8)	Sampling strategies CHS: a cohort that has been followed yearly since enrolment in 2002–2003, when participants were in kindergarten or first grade, from entire classrooms in schools in 12 communities throughout southern California H&H: Approximately 40 public high schools in the Los Angeles metropolitan area were approached about participating in this study. These schools were chosen because of their diverse demographic characteristics and proximity. Ten schools agreed to participate in the study, YASS: six schools from different DRG’s in Connecticut were invited to participate; of these, four agreed to participate.	Pen and paper	e-cigarette	Ever use of use-cigarette at baseline and ever use of tobacco at follow-up	Baseline measures of ever cigar use, ever e-cigarette use, grade, gender, race/ethnicity (White, Hispanic, Other), and site (CHS, H&H, YASS).
Leventhal 2015 Los Angeles California United States [42]	NR	Longitudinal cohort study, 3 time points, Follow up: 6 months	n analysed = 2530, retention rate: 97·0% at 6 months, 96·6% at 12 months	Male: 46·8%, Female: 53·2%, Age: 9th graders, ethnicity: American Indian n = 21 (0·8), Asian: n = 472 (19·0), Black: n = 119 (4·8), Native Hawaiian: n = 89 (3·6), White: n = 404 (16·2), Other: n = 142 (5·7), Multiethnic: n = 141 (5·7)	Ten public high schools in Los Angeles, California, were recruited through convenience sampling	Pen and paper	e-cigarette	Ever use of e-cigarette at baseline and cigarettes at follow-up AND other tobacco products at follow-up (hookah, cigars, any combustible products)	Age, sex, ethnicity, lives with biological parents, substance use, family history of smoking, parental education, peer smoking, depressive symptoms, impulsivity, delinquency, smoking susceptibility and expectancies.
Loukas 2018 Texas United States [43]	Marketing and Promotions across Colleges in Texas project (Project M-PACT).	Longitudinal design, 2 time points, Follow up: every 6 months for three waves (around 1 ·5 years)	n analysed: 2558, retention rates: 90·2% at wave 2 (*n* = 2,307), 89·1% at wave 3 (*n* = 2,279), and 91·8% at wave 4 (*n* = 2349)	Male: 32·3%, Female: 67·7%, Age: 18–25 (mean age 19), ethnicity: 31·8% were non-Hispanic white, 27·4% were Hispanic/Latino, 23·4% were Asian, 9·8% were African-American/Black, and 7·5% were another race/ethnicity or reported two or more races/ethnicities.	The sample were students involved in the first four waves of the Marketing and Promotions across Colleges in Texas project (Project M-PACT). Project M-PACT is a rapid response surveillance study, collecting data every six months from a cohort of 5,482 students attending one of 24 colleges in Texas.	Online	e-cigarette, vape pen, or e-hookah consistent with question	Ever use of e- cigarette and ever use of cigarette	Age, sex ethnicity, school type, cigarette susceptibility, family of origin tobacco use, friend cigarette use, ever other tobacco use
Lozano 2017 Mexico City, Guadalajara, and Monterrey Mexico [44]	NR	Longitudinal cohort study, 2 time points, follow-up: 20 months	n analysed: 4695, retention rate: 63%	Male: 48%, Female: 52%, Age at BL: 11–12 years (33%), >13 years (67%), Parental education: Primary: 16 Secondary: 38 High school: 19 University: 19 Unknown: 8	Sixty public middle schools from the three largest cities in Mexico (Mexico City, Guadalajara, and Monterrey) were selected using a stratified, multi-stage random sampling scheme.	Self-administered	e-cigarette	Ever use of e-cigarette use at baseline and ever use of tobacco at follow-up	Sex, age, parent socioeconomic status sensation seeking, friends that smoke, parents that smoke, siblings that smoke, tried alcohol, binge drinking and internet tobacco product advertising
Miech 2017 United States [45]	Monitoring the Future study	Longitudinal prospective cohort study, 2 time points, follow-up: 13·4 months	n analysed: 347, retention rate: 42%	Male: 47·6%, Female: 52·4%, Age: NR, Ethnicity: Non-white: 39·9	The target sample is all schools in the contiguous United States that enrol 25 or more 12th grade students, and in 2014 the study surveyed 122 schools (105 public and 17 private). The geographic areas sampled included the 28 largest metropolitan areas containing about one third of the nation’s population, as well as 136 other primary areas. Every year a random subsample of 2,450 members of the 12th grade class is selected to participate in a panel that receives follow-up surveys.	Pen and paper	e-cigarette	Current e-cigarette use at baseline and ever tobacco use at follow-up	Sex, race, and parental education
Morgenstern 2018, Lower Saxony and Schleswig-Holstein Germany [46]	NR	Longitudinal cohort study, 2 time points, follow up: 6 months	n analysed: 2,186, retention rate: 92·7%	Male: 47·9%, Female: 52·1% non-smokers and completed at follow up; Age: Range: 14–18 years	The data were obtained from a cluster-randomized study evaluating a school-based binge drinking prevention program. A total of 61 schools with 196 classes of 10th-grade students in the federal states of Lower Saxony and Schleswig-Holstein were included.	Pen and paper	e-cigarette	Ever use of e-cigarette use at baseline and ever use of tobacco at follow-up	Sex, age, state, school type, migration background, parents school leaving qualification, SES, personality traits (multiple), substance consumption (5 substances) and intervention
Osibogun 2020 United States [47]	Population Assessment of Tobacco and Health Study (PATH)	Prospective cohort study, 2 time points, follow-up: 24 months	n analysed: 6,523, retention rate: NR	Male: 52%, Female: 48% Age range: 12–17 years; ethnicity: White 47·1%, African American 14%, Hispanic 29·6%, other 9·3%	This longitudinal survey’s cohort was selected via a 4-stage, stratified probability sample that was nationally representative.	audio computer-assisted self-interviewing.	e-cigarette	Current e-cigarette use at baseline and current cigarette use at follow-up	Age, sex, ethnicity, parents education level, other tobacco products, lives with tobacco user, noticed health warnings, risk taking
Penzes 2018 Tirgu Mures Romania [48]	NR	Longitudinal cohort study, 2 time points, follow-up: 6 months	n analysed: 1,369, retention rate: 68·4%	Male: 45·7%, Female: 54·3%, mean age: control group 14·9 (0·5) years and intervention 14·9 (0·5), 52·4% Romanian, 72% high grades (academic achievement)	The sampling frame included all 9th grade students in the 16 high schools of the city (Tirgu Mures, Romania). three classes from one school declined participation,	Online	e-cigarette	Ever use of e- cigarette at baseline and ever use of cigarette at follow-up AND ever use of waterpipe at follow-up	Intervention/control condition, gender, and age were included in the analyses
Primack 2015 United States [49]	Dartmouth Media, Advertising, and Health Study	Longitudinal cohort study, 2 time points, Follow up: approximately 12 months	n analysed: 626, retention rate: 69·6%	Male: 46·1%, Female: 53·9%, Age: 18–26 (mean age <20), ethnicity: non-Hispanic white n = 531 (76·5%)	Data come from the second and third waves of the United States–based Dartmouth Media, Advertising, and Health Study, a national study of adolescents and young adults (aged 16–26 years) recruited via random digit dialling using landline (66·7%) and cellular telephone numbers (33·3%).	Online	e-cigarette	Ever use of e-cigarettes at baseline and ever use of tobacco at follow-up	Age, sex, race/ethnicity, maternal education level, sensation seeking tendency, smoking
Spindle 2017 Richmond Virginia United States [50]	Spit for Science (S4S) project	Longitudinal cohort, 2 time points, Follow up: 12 months	n analysed = 2316, retention rate: 70%	Male/Female: % NR, Age: 18 or older (mean age 18·5), ethnicity: White: 47%; Black: 19%; Asian: 17%; Hispanic/Latino: 6%, mixed race/ethnicity: 7%	The sample for the current study was a subset of the Spit for Science (S4S) project, a university-wide longitudinal study aimed at assessing genetic and environmental influences on substance use and emotional health in college students.	Online	e-cigarette	Ever use of e-cigarette at baseline and ever use of tobacco at follow-up; Current e-cigarette use at baseline and ever tobacco use at follow-up; Current e-cigarette use at baseline and current tobacco use at follow-up	Gender, age, race/ethnicity, impulsivity (all five subscales), depression/anxiety, stressful life events, peer deviance, and ever use of other tobacco products were included as covariate
Treur 2018 Netherlands [51]	NR	Longitudinal quasi-experimental study, 2 time points, follow-up: 6 months	n analysed: 2,100, retention rate: NR	Male: 51·8, Female: 48·2, Age: mean age = 13·8 (SD = 1·1)—all Cohort I participants not just out of 2100 included in longitudinal analysis, ethnicity NR	Nineteen secondary schools chosen based on their current smoking policy, and future intentions to implement an outdoor smoking ban identified by a national monitor questionnaire for a quasi-experimental study	Mixed	ENDS, ENNDS	Ever ENNDS use at baseline and current tobacco use at follow-up	Sex, age, educational level, propensity to smoke, intervention
Watkins 2018 United States [52]	Population Assessment of Tobacco and Health Study (PATH)	Longitudinal prospective cohort study, 2-time points, follow-up: 1 year	Follow up: n = 10, 384, retention rate: 87·9%	Male: 50·9%, Female: 49·1%, mean age: 14·3 (1·7) years, range 12–17 years, 52·5% white, 13·9% African American, 22·3% Latino, 11·3% other	A 4-stage, stratified probability sample design. Adults (age ≥18 years, up to 2 per household) were oversampled for tobacco users, African American individuals, and young adults (age 18–24 years). The PATH youth sample consists of individuals whose parents were sampled for the PATH adult survey. Up to 2 youths were selected per household; sample and replicate weights were generated so that the sampled population reflected the non-institutionalized youth population at baseline.	In-person computer-assisted interviews at home.	e-cigarette	Ever use of e-cigarettes at baseline and ever use of tobacco at follow-up;Current ENDS/ENNDS use at baseline and ever tobacco use at follow-up;Current ENDS/ENNDS use at baseline and current tobacco use at follow-up	Model includes all ever tobacco use categories and the following wave 1 covariates: female, age, race/ethnicity, parental educational level, sensation seeking, alcohol ever use, living with tobacco user, notice of cigarette warning labels, tobacco advertising receptivity, and summer season
Wills 2017 Oahu Hawaii United States [53]	NR	Longitudinal prospective cohort study, 2 time points, follow-up: 12 months	Analysed: 1136, consent rate: 70% and 67% at follow-up	Male/Female% NR, Age: Grades 9–10 at baseline, ethnicity NR	Six high schools (four public and two private) on Oahu, Hawaii. The sampling frame was all students in the 9th and 10th grades with adequate English language ability.	Pen and paper	e-cigarette	use of e-cigarette at baseline and cigarette use at follow up	NR

NR, not reported

**Fig 2:**
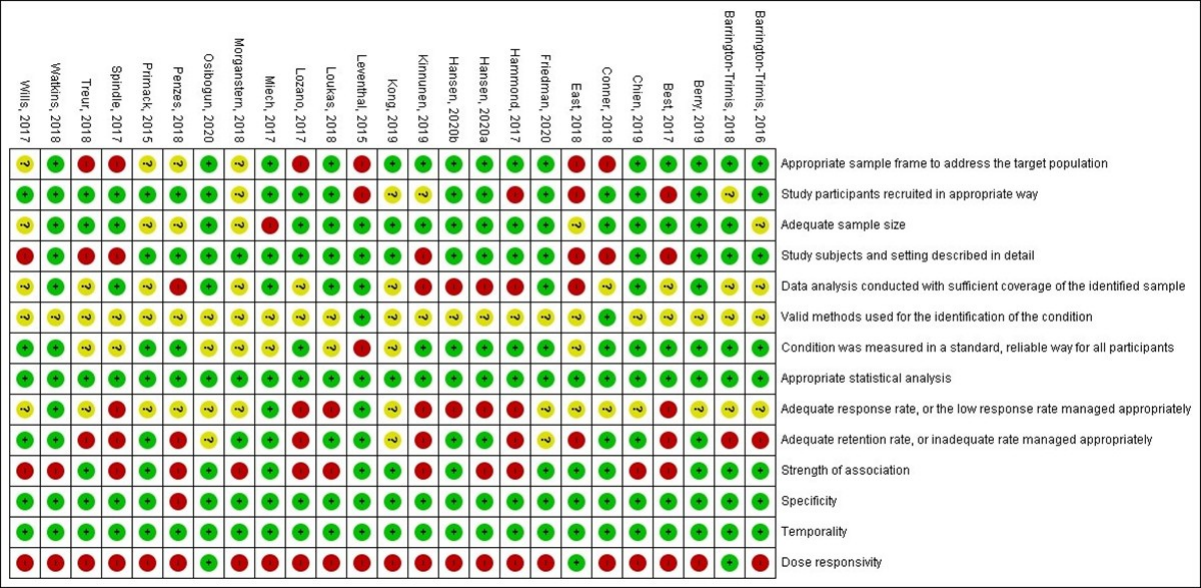
Risk of bias data.

Sixteen studies had high to moderate risk of bias (defined as meeting less than 7 of the 11 risk of bias criteria), [33, 34, 36–41, 44, 46–51, 53] while nine had a low risk (defined as meeting 7 or more criteria). [17, 18, 35, 42, 43, 45, 52, 54, 55] (See Fig 2). Key methodological issues identified in the studies were the sampling frame was not appropriately representative of the target population (n = 6 studies rated as high risk [37, 38, 42, 44, 50, 51] and 4 studies rated as unclear [46, 48, 49, 53]), lack of use of valid methods to identify the condition (n = 23 used self-reported measures without established psychometrics and were rated unclear [17, 18, 33–36, 38–41, 43–55]), and lack of information regarding whether the response rate was adequate or appropriately managed (n = 8 studies rated as high risk [18, 36, 39, 40, 43, 44, 50, 55] and 22 studies rated as unclear [17, 33–35, 37, 38, 41, 46–49, 51, 53, 54]). All 25 studies were rated as low risk on the criteria for appropriate statistical analysis and 18 were also rated as low risk for adequate sample size.[17, 18, 34–37, 39–44, 47, 50–52, 54, 55] For the Bradford-Hill criteria, 13 studies met ≥ three of the four criteria. [33–35, 37, 38, 41, 42, 45, 47, 49, 51, 54, 55]. All studies rated low risk for temporality, and most were also rated low risk for specificity (n = 24). Only three studies were rated low risk for the dose responsivity criteria, [34, 38, 47]. The majority of studies met the criteria for specificity and all met the criteria for temporality. All studies except one included in this review reported a positive association, with 13 reporting an adjusted odds ratio of > 4.

**Fig 3:**
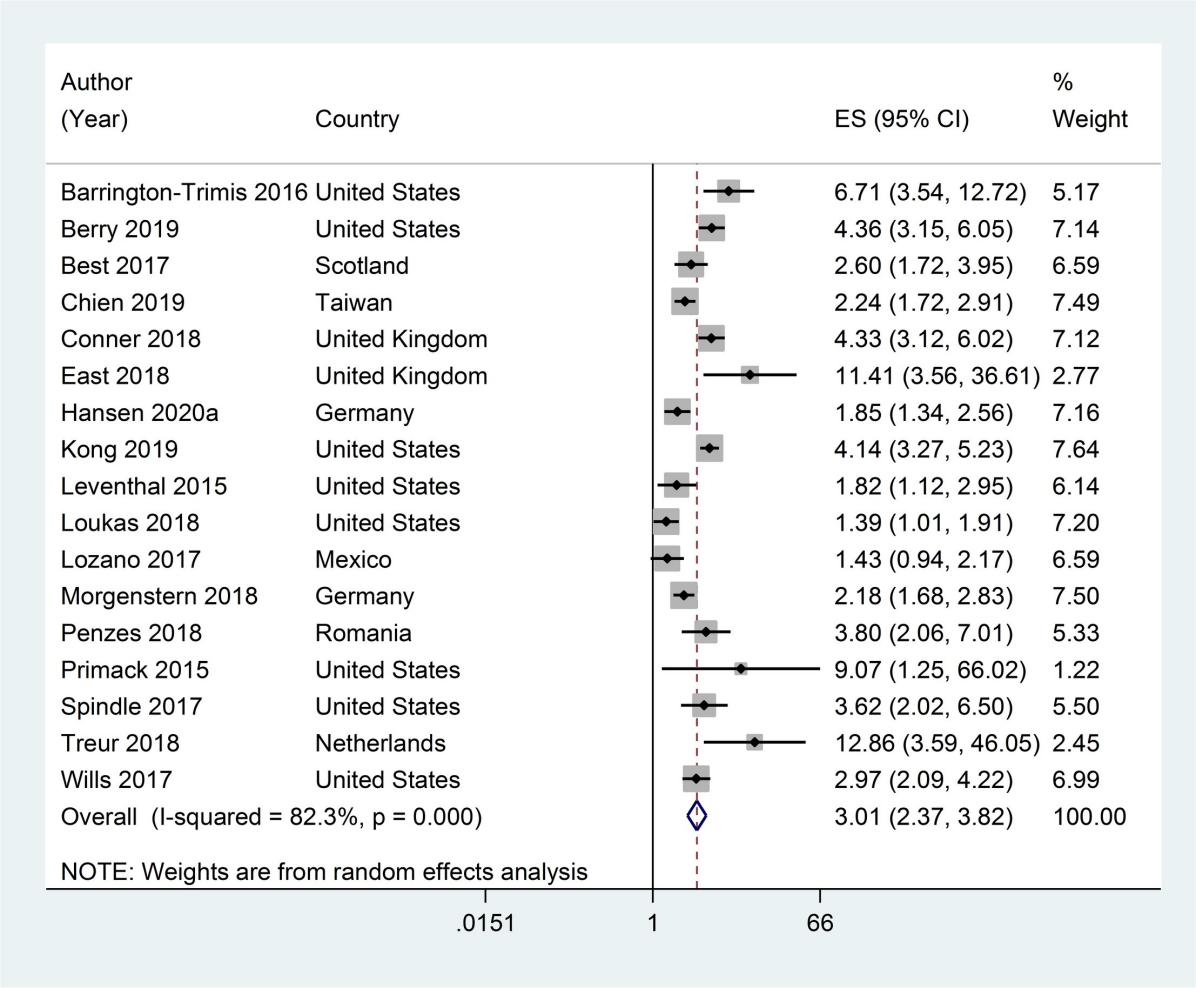
Forest plot of adjusted risk ratios assessing the association between ever e-cigarette use at baseline and subsequent ever cigarette use at follow-up.

Seventeen studies assessed the association between ever ENDS/ENNDS use and subsequent ever cigarette use. [17, 18, 33, 35–38, 41–44, 46, 48, 49, 51, 53] The adjusted RRs ranged from 1·39 (95% CI: 1·01, 1·91) to 12·86 (95% CI: 3·59, 46·05); with a pooled RR of 3·01 (95% CI: 2·37, 3·82, p<0·001; I^2^ = 82·3%, p <0·001) (see Fig 3). Most studies adjusted for covariates including sex and age or grade (n = 15), with the majority (n = 14) also adjusting for additional variables including susceptibility to smoking, influence by friends and family, psychological constructs and status, and exposure to advertising.

**Fig 4:**
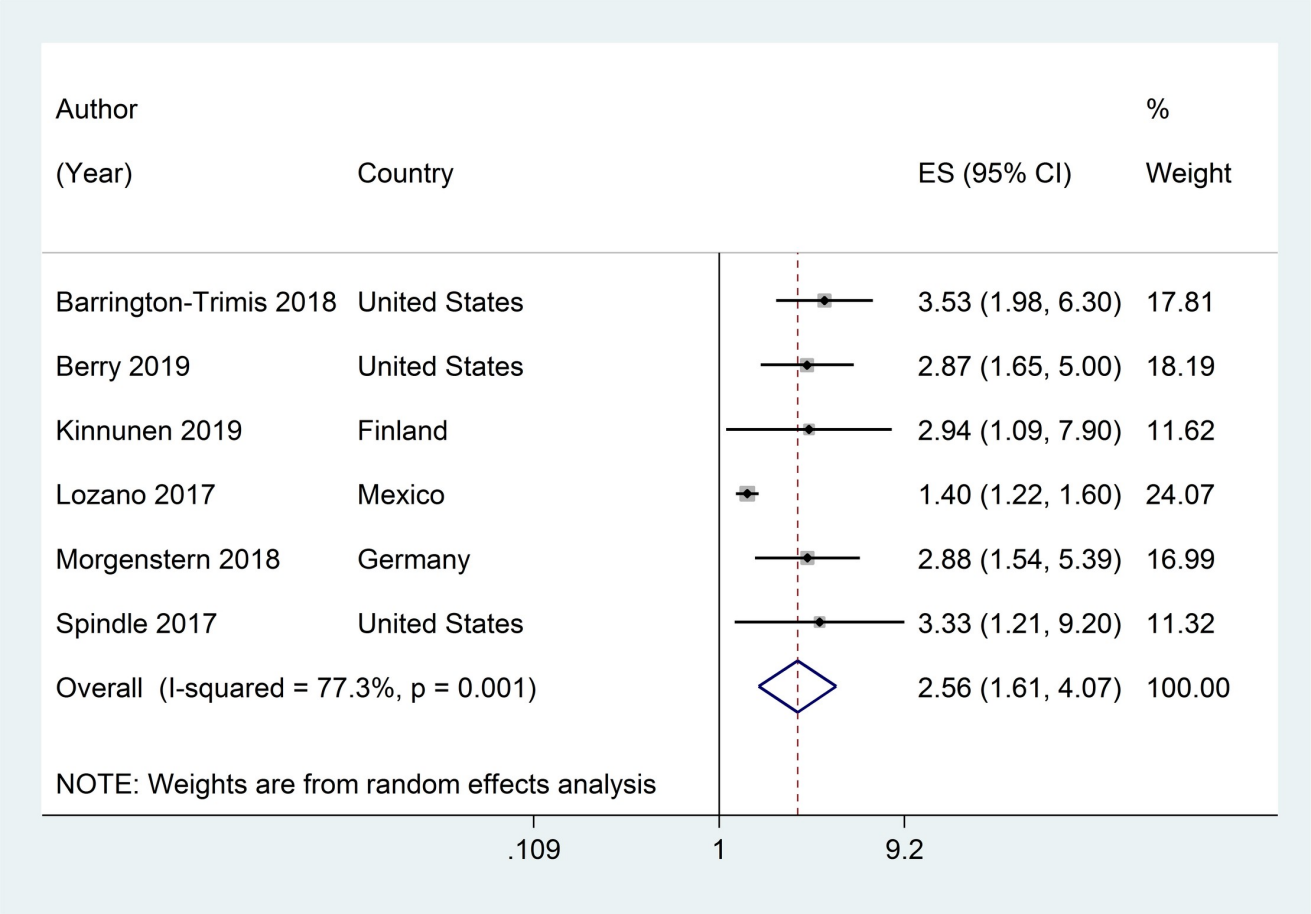
Forest plot of adjusted risk ratios assessing the association between ever e-cigarette use at baseline and subsequent current cigarette use at follow-up.

Six studies assessed the association between ever ENDS/ENNDS use at baseline and subsequent current cigarette use at follow-up. [34, 35, 40, 44, 46, 50] The adjusted RRs ranged from 1·40 (95% CI: 1·22, 1·60) to 3·53 (95% CI: 1·98, 6·30); with a pooled RR of 2·56 (95% CI: 1·61, 4·07, p<0·001; I^2^ = 77·3%, p = 0·001) (see Fig 4).

**Fig 5:**
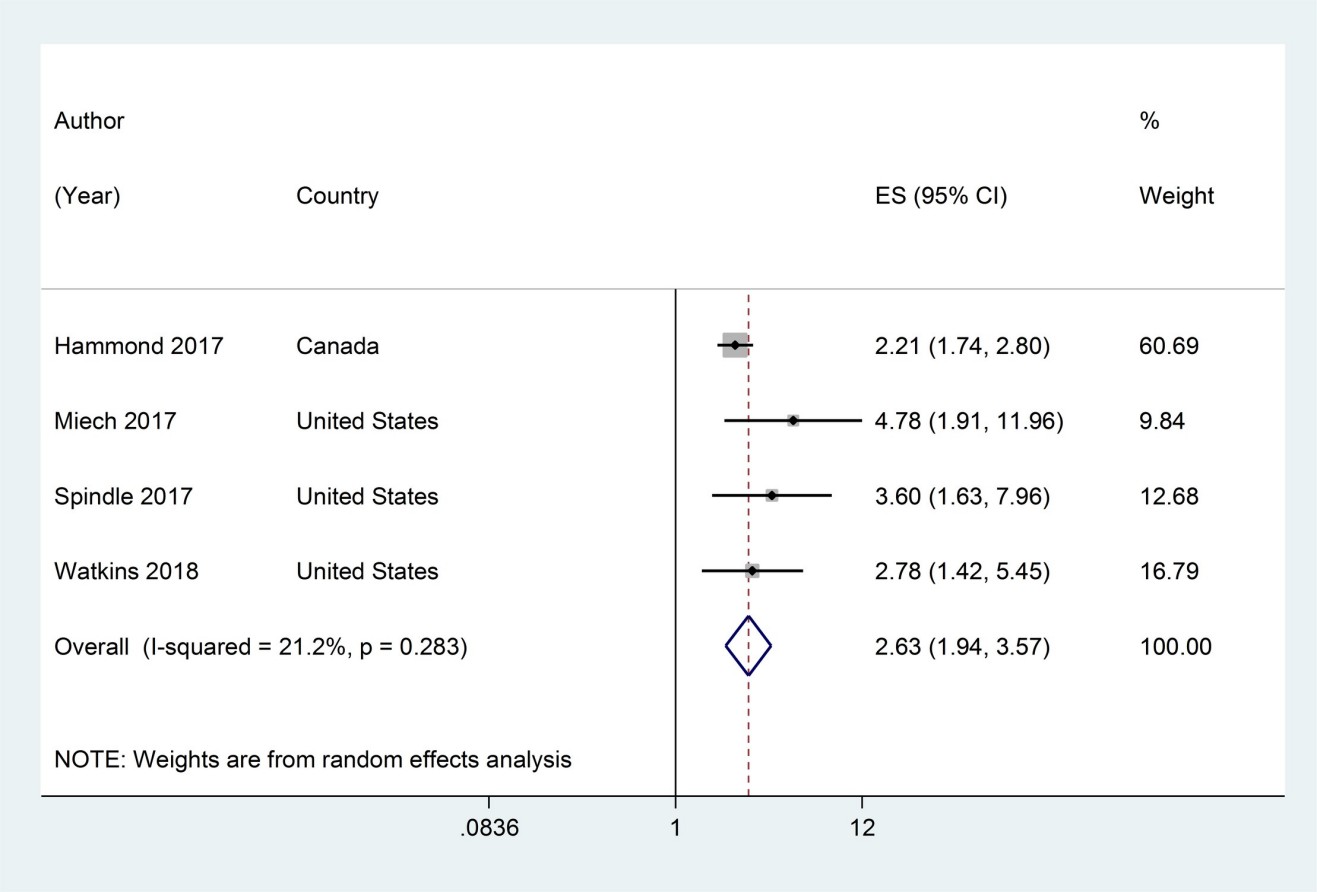
Forest plot of adjusted risk ratios assessing the association between current e-cigarette use at baseline and subsequent ever cigarette use at follow-up.

Four studies assessed the association between current ENDS/ENNDS use at baseline and subsequent ever cigarette use at follow-up. [39, 45, 50, 52] The adjusted RRs ranged from 2·21 (95% CI: 1·74, 2·80) and 4·78 (95% CI: 1·91, 11·96) with a pooled RR of 2·63 (95% CI: 1·94, 3·57, p<0·001; I^2^ = 21·2%, p >0·05) (see Fig 5).

**Fig 6:**
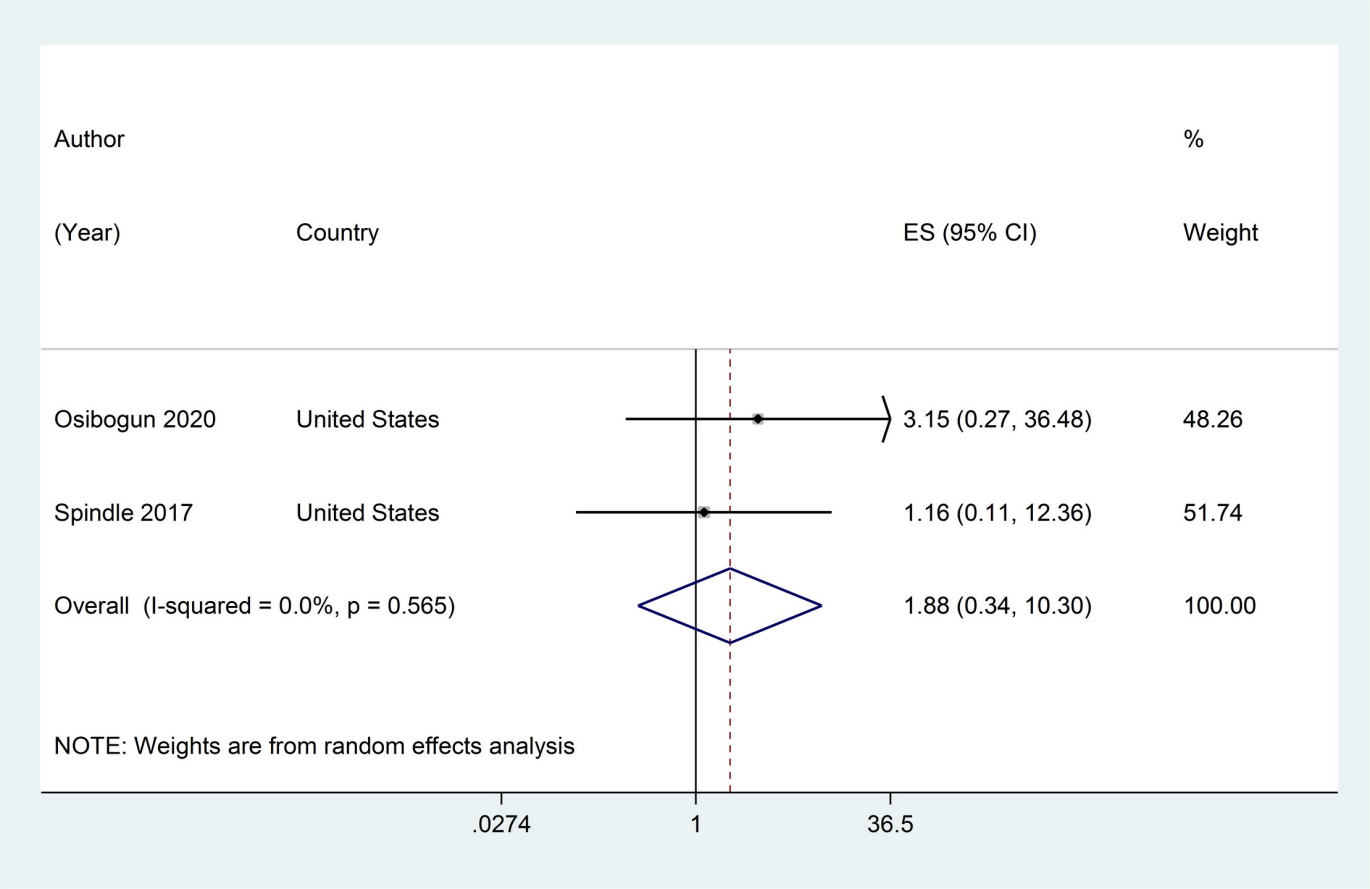
Forest plot of adjusted risk ratios assessing the association between current e-cigarette use at baseline and subsequent current cigarette use at follow-up.

Two studies assessed association between current ENDS/ENNDS use at baseline and subsequent current cigarette use at follow-up. [47, 50] The adjusted RRs were 1·16 (95% CI: 0·11, 12·36) and 3·15 (95% CI: 0·27, 36·48), with a pooled RR of 1·88 (95% CI: 0·34, 10 ·30, p = 0·467; I^2^ = 0%, p >0·05) (see Fig 6).

S3 Table lists the four studies that assessed the association between ENDS/ENNDS use at baseline and subsequent use of other tobacco products including hookah, cigar, pipe, and other tobacco products at follow-up, where significant associations were reported.

**Fig 7:**
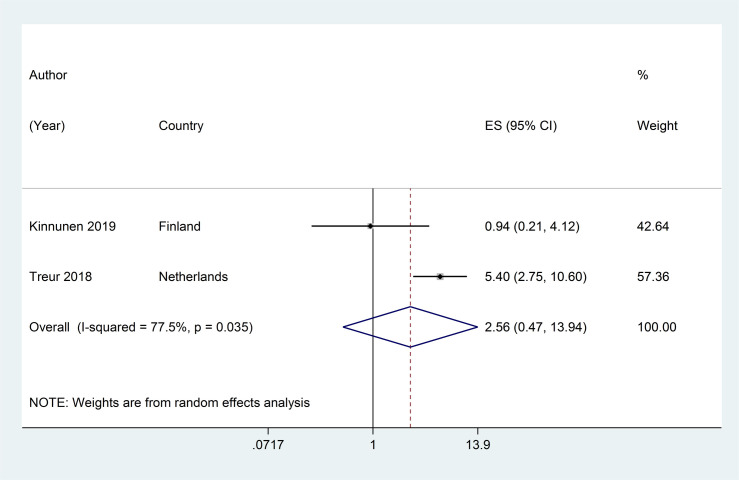
Forest plot of adjusted risk ratios assessing the association between ever ENNDS use at baseline and subsequent current or ever cigarette use at follow-up.

Only two studies [40, 51] assessed the association between ENNDS use at baseline and subsequent cigarette use (current or ever) at follow-up. The pooled adjusted RR of 2.56 (95% CI: 0·47, 13·94, I^2^ = 77·5%, p = 0.277;) (see Fig 7). No study reported on association between ENNDS use with subsequent use of other tobacco products.

The unadjusted estimates are available as supplementary materials (S2 and S3 Tables, S1–S3 Figs).

One study [54] reported no difference in uptake of ENDS/ENNDS use at follow up between flavoured vs unflavoured e-cigarette use at baseline (RR: 0·24 (95% CI 0·05, 1·0) when controlling for sex, age, state, school type, migration background, parent’s qualifications, socioeconomic status (SES), multiple personality traits, and consumption of five substances.

The adjusted RRs were similar by geographic location, year of publication, and length of follow up (see S4A–S4C Fig). There were some differences in effect sizes by study quality, with higher quality studies reporting lower adjusted RRs (risk of bias ≥7 (higher quality): 2·16 (95% CI: 1·47, 3·16, p<0·001; I^2^ = 85·0%, p <0·001) compared to lower quality studies (risk of bias scores <7: 3·57 (95% CI: 2·69, 4·73, p<0·001; I^2^ = 76·9%, p < ·001)) see S4D Fig. Studies that scored > = 3 on the Bradford-Hill criteria for causal inference had higher adjusted RRs of 4·47 (95% CI: 3·28, 6·09, p<0·001; I^2^ = 65·0%, p = 0·006) relative to studies that scored <3: 2·21 (95% CI: 1·80, 2·70, p<0·001; I^2^ = 64·1%, p = 0·004) (see S4E Fig).

The adjusted RRs for baseline ever ENDS/ENNDS use and current cigarette use at follow-up were similar by geographic location, year of publication, length of follow up, study quality, and score for Bradford-Hill causal inference (S5A–S5E Fig).

We did not undertake subgroup analysis examining other associations due to the small number of studies included in the main meta-analyses (four or less).

**Fig 8:**
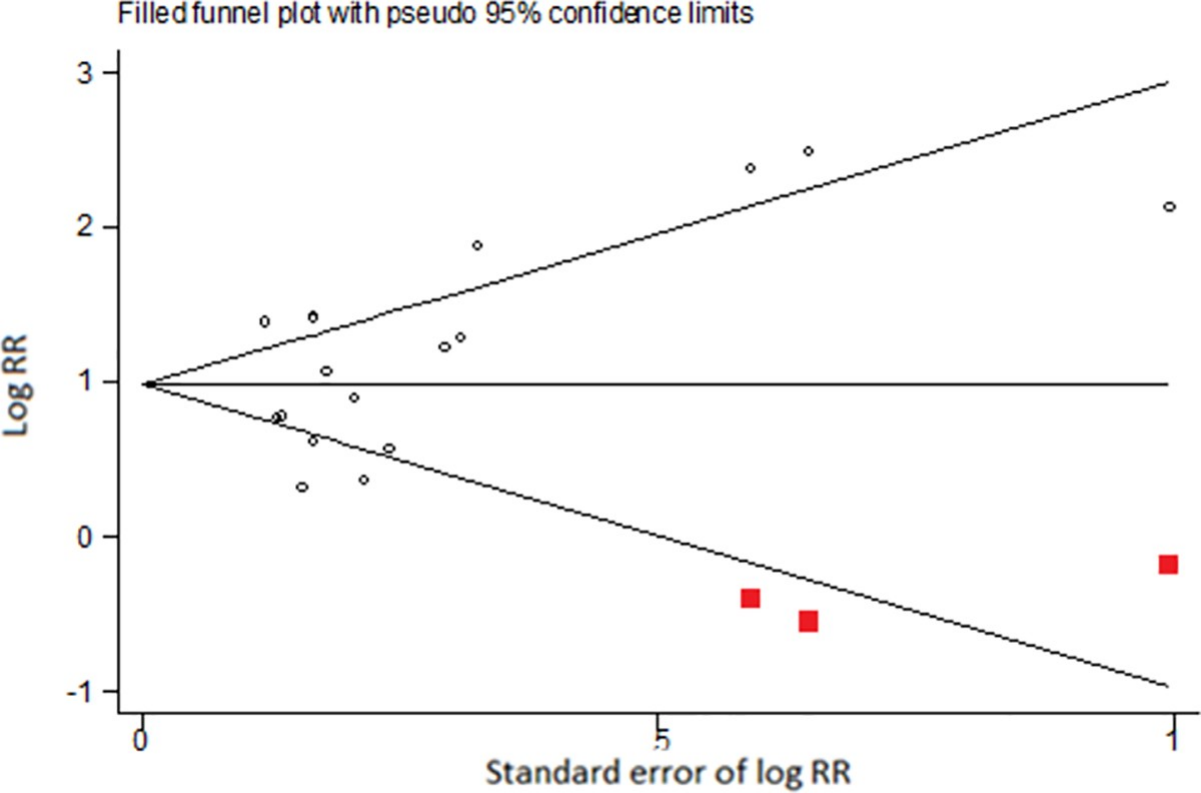
Funnel plot illustrating results from trim-and-fill analysis of adjusted log RRs for outcome ever e-cigarette use at baseline and ever cigarette use at follow-up.

For ever ENDS/ENNDS use at baseline and ever cigarette use at follow-up the adjusted results, three studies were estimated as missing due to funnel plot asymmetry. Results from the trim-and-fill analysis found that the bias-adjusted pooled RR was 2·75 (95% CI: 2·16, 3·49), which was only slightly lower than the adjusted pooled RR from the primary analysis (see Fig 8).

**Fig 9:**
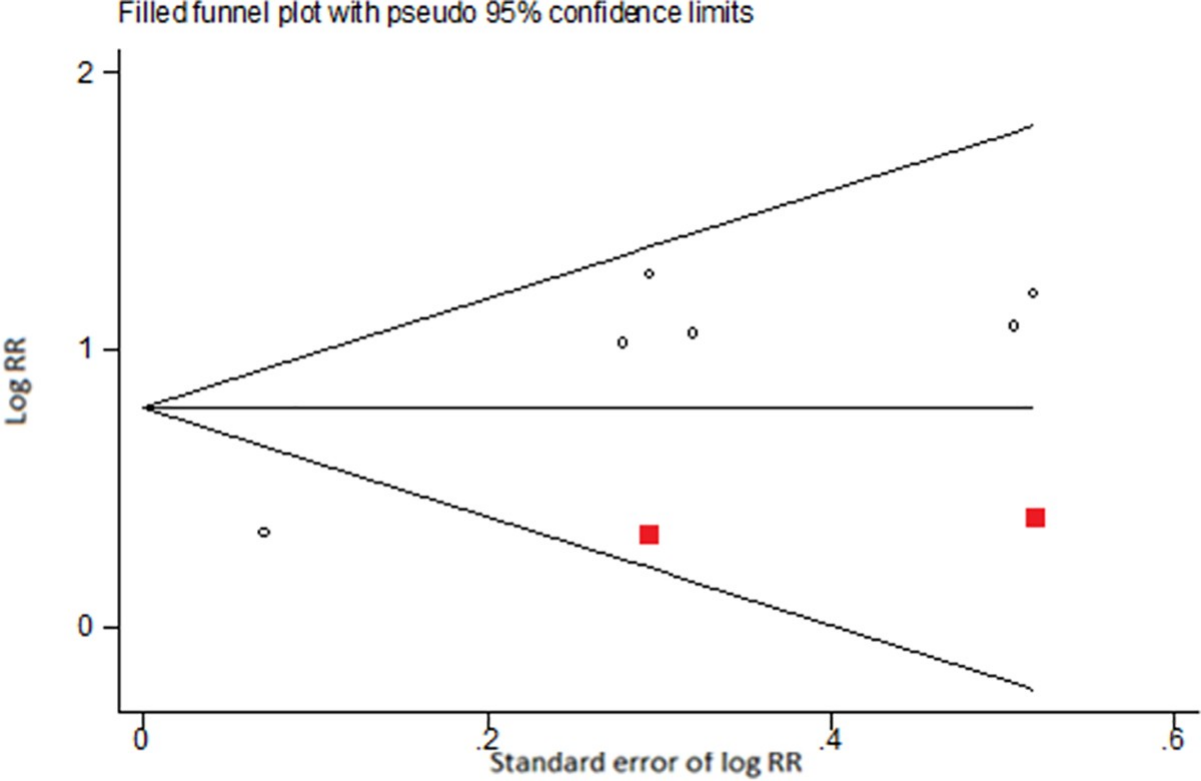
Funnel plot illustrating results from trim-and-fill analysis of adjusted log RRs for outcome ever e-cigarette use at baseline and current cigarette use at follow-up.

For ever ENDS/ENNDS use at baseline and current cigarette use at follow-up, the adjusted results two studies were estimated as being missing due to funnel plot asymmetry. Results from the trim-and-filled analysis found that the bias-adjusted pooled RR was 2·21 (95% CI: 1·55, 3·17), which was slightly lower than the original estimate (see Fig 9).

## Discussion

This review supports evidence of a longitudinal association between ENDS/ENNDS use at baseline and subsequent tobacco use in those aged <20 years. Studies included in the meta-analysis found a significant positive adjusted association between ever ENDS/ENNDS and current cigarette use (2·56 (95% CI: 1·61, 4·07) at follow-up among children and adolescents aged <20 years. A positive association was also found between current e-cigarette use and current cigarette use at follow-up (RR: 1·88 (95% CI: 0·34, 10·30)), and ENNDS use at baseline and later cigarette use (RR: 2·56 (95% CI: 0·47, 13·94)). Despite the relatively large effect size, evidence of these associations was not statistically significant potentially due to the small number of studies included, and thus require further exploration in prospective studies.

Our findings are similar, albeit slightly weaker, to those reported by Khouja et al, [12] where a significant association between e-cigarette use among non-smokers and later tobacco smoking was found. The similarity may, in part, be due to the inclusion of many of the same studies. However, our review included more recently published studies, included a broader representation of study locations outside of the US (13/25 studies), focused entirely on children and adolescents (whereas the review by Khouja et al. included those up until the age of 30) [12], and excluded case-control studies that are at risk of increased bias. Consequently, this study has improved both the robustness, precision of aggregate analysis and international applicability of findings from prior reviews.

In our exploratory subgroup analysis, we found that higher quality studies had small estimates than lower quality studies. The impact of different methodological biases have been explored in a recent review examining the association between e-cigarette use and initiation of conventional cigarette use. [15] This review described potential bias relating to attrition, where studies that reported on findings from complete case analyses found larger effect sizes than when imputed data was included. Additionally, studies that adjusted for a more comprehensive list of known confounders also reported smaller estimates, compared to those that adjusted for fewer confounders. [15, 56] Future studies need to better consider and address such methodological differences to provide better estimates of the association between e-cigarette use and conventional cigarette uptake. All but one of the studies included in this review reported a positive association (RR>1) between ENDS/ENNDS use and future cigarette use among children and adolescents. The only industry-funded study that met the eligibility criteria for this review was excluded from the meta-analysis due to overlap of data with other studies. The authors of this study undertook various sensitivity analysis adjusting for multiple confounders. [56, 57] Whilst the authors concluded that adjustment for various confounders including propensity to smoke reduced the strength of the association, all adjusted odds ratios were larger than one, consistent with findings from non-industry sponsored studies.

Our review found evidence of a consistent positive association between ENDS/ENNDS use and cigarette smoking across a large number of studies internationally. This provides strong evidence to support the causal relationship between ever ENDS/ENNDS and ever smoking for this age group. These findings are of concern as other cross-sectional studies have reported that children and adolescents who use ENDS and/or ENNDS have different psychological profiles to current smokers, and would have otherwise have been at low risk of smoking. [58–61] As such, there is an urgent need for governments internationally to take action to regulate the availability and marketing of ENDS/ENNDS products to children and adolescents.

Further, the US Surgeon General’s Report concluded that ENDS/ENNDS were unsafe for use among children and adolescents due to a range of health-related adverse effects. [62] The use of ENDS/ENNDS may also contribute to increased burden of tobacco-related harms on individuals and communities. [63] In part due to such an association, modelling weighing the potential health benefits (e.g. cessation among established smokers) and harms associated with e-cigarettes found, overall, that ENDS/ENNDS use would yield a net harm and lead to 1,510,000 years life lost in the US. [63] This modelling is based on results from a single clinical trial of ENDS/ENNDS provided as part of medically-supervised cessation benefits Such findings are consistent with later reviews of randomised trials assessing the use of ENDS/ENNDS, [64, 65] however presents an overestimation of benefit when used as consumer products in the general population. As presented in a synthesis of observational studies, there are no apparent population-level increase in cessation when using e-cigarettes as a consumer product [65].

Given such considerations, a report by WHO provides a range of policy options including a ban on their sale; product taxation; and preventing the use of ENDS/ENNDS indoors and in areas to prevent use in in children and adolescents but also uptake in adults more broadly [66, 67]. These are supported by recommendations and policy statements nationally and internationally.[67, 68] Given the susceptibility of children and adolescents to marketing and the appeal of flavouring, governments should restrict all forms of promotion and marketing to children and adolescents and ban all characterising flavours. [68, 69] A number of recently published studies have also reported promising findings regarding the impact of local retail regulations, [70] and the prohibition of the sale of flavoured products on ENDS/ENNDS use in youth, [71] however, rigorous evaluation of the impacts of comprehensive policy approaches is warranted. Early findings from two studies suggest mixed findings between ENNDS and cigarette smoking. Whilst, still inconclusive, precautionary principles should be in place when considering the regulation for all forms of e-cigarettes, including those that do and do not contain nicotine.

There were few studies that measured association between current ENDS/ENNDS and current cigarette use. Further studies are needed to establish whether current ENDS/ ENNDS result in current cigarette given this Similarly, there were few studies assessing the impact of non-nicotine and flavoured tobacco products, and as such any conclusions need to be interpreted in light of this. Most studies were conducted in high-income countries. Consequently, the study results may be limited in their generalisability. The data from included studies may also be subject to social desirability and other reporting biases due to the self-report nature of the data collection methods. There was high heterogeneity in the meta-analysis, unexplained by the subgroup analysis, indicating that the reasons for the variation remains unknown. The trim and fill funnel plots suggest there may be some publication bias, but the bias-adjusted estimates were similar to those calculated from the main analysis. Finally, despite efforts to select outcomes that controlled for pre-specified confounders, restricting outcomes that controlled for these confounders only was not always possible. Consequently, there were differences between studies in terms of the characteristics that were controlled for, which may contribute to the high level of heterogeneity.

Nonetheless, the findings provide consistent evidence from observational studies of an association between ENDS/ENNDS use among non-smoking children and adolescents, and subsequent tobacco use, in particular cigarettes. Government regulation and implementation to prevent use of ENDS/ENNDS among youth however varies considerably globally. [69, 72] The experience of global efforts to combat the use of conventional cigarettes and other tobacco products suggests that such efforts are inadequate to sufficiently avert the projected harms, if the current trajectory continues. There is a need for countries internationally to prioritise the adoption and implementation of comprehensive measures as outlined in the WHO Framework Convention on Tobacco Control to prevent uptake of ENDS/ENNDS and regulates availability in children and adolescents, up to imposing a ban, to prevent uptake of ENDS/ENNDS for this group.

## Supporting information

S1 Checklist (DOC)

S1 Fig Forest plot of unadjusted risk ratios assessing the association between ever e-cigarette use at baseline and ever cigarette use at follow-up. (DOCX)

S2 Fig Forest plot of unadjusted risk ratios assessing the association between ever e-cigarette use at baseline and current cigarette use at follow-up. (DOCX)

S3 Fig Forest plot of unadjusted risk ratios assessing the association between current e-cigarette use at baseline and ever cigarette use at follow-up. (DOCX)

S4 Fig (A) Forest plot of adjusted risk ratios assessing the association between ever e-cigarette use at baseline and ever tobacco use at follow-up by country. (B) Forest plot of adjusted risk ratios assessing the association between ever e-cigarette use at baseline and ever tobacco use at follow-up by year of publication. (C) Forest plot of adjusted risk ratios assessing the association between ever e-cigarette use at baseline and ever cigarette use at follow-up by length of follow-up. (D) Forest plot of adjusted risk ratios assessing the association between ever e-cigarette use at baseline and ever cigarette use at follow-up by overall risk of bias score. (E) Forest plot of adjusted risk ratios assessing the association between ever e-cigarette use at baseline and ever cigarette use at follow-up by Bradford Hill’s criteria for causal inference.
(DOCX)

S5 Fig (A) Forest plot of adjusted risk ratios assessing the association between ever e-cigarette use at baseline and current tobacco use at follow-up by country. (B) Forest plot of adjusted risk ratios assessing the association between ever e-cigarette use at baseline and current tobacco use at follow-up by year of publication. (C) Forest plot of adjusted risk ratios assessing the association between ever e-cigarette use at baseline and current tobacco use at follow-up by length of follow-up. (D) Forest plot of adjusted risk ratios assessing the association between ever e-cigarette use at baseline and current tobacco use at follow-up by overall risk of bias score. (E) Forest plot of adjusted risk ratios assessing the association between ever e-cigarette use at baseline and current tobacco use at follow-up by risk of bias score for causal inference.
(DOCX)

S1 Table Additional Bradford-Hill causal inference criteria. (DOCX)

S2 Table Unadjusted and adjusted risk ratios for association between ENDS/ENNDS and cigarette use. (DOCX)

S3 Table Unadjusted and adjusted risk ratios for association between ENDS/ENNDS and other tobacco products. (DOCX)

S1 Appendix Search strategy. (DOCX)

S1 Data (XLS)
