# Supplementary material for: Association between electronic nicotine delivery systems and electronic non-nicotine delivery systems with initiation of tobacco use in individuals aged < 20 years. A systematic review and meta-analysis
Source: PLoS One. 2021 Sep 8;16(9):e0256044. doi: 10.1371/journal.pone.0256044 (PMC8425526; doi:10.1371/journal.pone.0256044)
Supplement: S2 Fig — (DOCX) [file pone.0256044.s003.docx]

**S2 Figure. Forest plot of unadjusted risk ratios assessing the association between ever e-cigarette use at baseline and current cigarette use at follow-up**
