# Supplementary material for: Association between electronic nicotine delivery systems and electronic non-nicotine delivery systems with initiation of tobacco use in individuals aged < 20 years. A systematic review and meta-analysis
Source: PLoS One. 2021 Sep 8;16(9):e0256044. doi: 10.1371/journal.pone.0256044 (PMC8425526; doi:10.1371/journal.pone.0256044)
Supplement: S4 Fig — (A) Forest plot of adjusted risk ratios assessing the association between ever e-cigarette use at baseline and ever tobacco use at follow-up by country. (B) Forest plot of adjusted risk ratios assessing the association between ever e-cigarette use at baseline and ever tobacco use at follow-up by year of publication. (C) Forest plot of adjusted risk ratios assessing the association between ever e-cigarette use at baseline and ever cigarette use at follow-up by length of follow-up. (D) Forest plot of adjusted risk ratios assessing the association between ever e-cigarette use at baseline and ever cigarette use at follow-up by overall risk of bias score. (E) Forest plot of adjusted risk ratios assessing the association between ever e-cigarette use at baseline and ever cigarette use at follow-up by Bradford Hill’s criteria for causal inference. (DOCX) [file pone.0256044.s005.docx]

**S4A Figure. Forest plot of adjusted risk ratios assessing the association between ever e-cigarette use at baseline and ever tobacco use at follow-up by country**

**S4B Figure. Forest plot of adjusted risk ratios assessing the association between ever e-cigarette use at baseline and ever tobacco use at follow-up by year of publication**

**S4C Figure. Forest plot of adjusted risk ratios assessing the association between ever e-cigarette use at baseline and ever cigarette use at follow-up by length of follow-up**

**S4D Figure. Forest plot of adjusted risk ratios assessing the association between ever e-cigarette use at baseline and ever cigarette use at follow-up by overall risk of bias score**

**S4E Figure. Forest plot of adjusted risk ratios assessing the association between ever e-cigarette use at baseline and ever cigarette use at follow-up by Bradford Hill’s criteria for causal inference**
