# Supplementary material for: Association between electronic nicotine delivery systems and electronic non-nicotine delivery systems with initiation of tobacco use in individuals aged < 20 years. A systematic review and meta-analysis
Source: PLoS One. 2021 Sep 8;16(9):e0256044. doi: 10.1371/journal.pone.0256044 (PMC8425526; doi:10.1371/journal.pone.0256044)
Supplement: S1 Table — (DOCX) [file pone.0256044.s007.docx]

**S1 Table: Additional Bradford-Hill causal inference criteria**

| **Criteria** | **Categories** | **Definition** |
| --- | --- | --- |
| Strength of Association | Strong association = Low risk  Weak association = High risk | Strong associations were defined as having an adjusted odds ratio of four or more (the actual effect estimate, not 95% CIs). Where available, this was assessed against the adjusted odds ratio for ever/current e-cigarette use at baseline and current cigarette use at follow-up. If not, it was assessed against the main analysis. |
| Specificity | Yes = Low risk  No = High risk | Studies needed to adjust for more than basic demographics (i.e. sex, age, socioeconomic position) to meet the specificity criterion. |
| Temporality | Yes = Low risk  No = High risk | Studies were required to measure use longitudinally (i.e., ENDS/ENNDS and cigarette use was measured at time point one with cigarette use measured subsequently at time point two) in order to meet the temporality criterion. |
| Dose responsivity | Yes = Low risk  No = High risk | Studies needed to measure and consider as part of the analysis the frequency of ENDS/ENNDS use, how long the product was used for, and/or the quantity of nicotine in the e-liquid used, in order to met the dose responsivity criterion. |
