# Supplementary material for: Association between electronic nicotine delivery systems and electronic non-nicotine delivery systems with initiation of tobacco use in individuals aged < 20 years. A systematic review and meta-analysis
Source: PLoS One. 2021 Sep 8;16(9):e0256044. doi: 10.1371/journal.pone.0256044 (PMC8425526; doi:10.1371/journal.pone.0256044)
Supplement: S2 Table — (DOCX) [file pone.0256044.s008.docx]

**S2 Table. Unadjusted and adjusted risk ratios for association between ENDS/ENNDS and cigarette use**

| **Outcome: *Current e-cigarette use at baseline and ever cigarette use at follow-up*** | | | |
| --- | --- | --- | --- |
| **Author (year)** | **Geographical area** | **Unadjusted RR**  **(95% CI)** | **Adjusted RR**  **(95% CI)** |
| Hammond 2017 | Canada | 5.11 (4.12, 6.32) | 2.21 (1.74, 2.80) |
| Miech 2017 | United States | 4.60 (1.71, 12.36) | 4.78 (1.91, 11.96) |
| Spindle 2017 | United States | Insufficient data to calculate | 3.60 (1.63, 7.96) |
| Watkins 2018 | United States | 3.81 (1.89, 7.68) | 2.78 (1.42) |
| **Outcome:** *Current e-cigarette use at baseline and current cigarette use at follow-up* | | | |
| Osibogun 2020 | United States | 5.09 (0.28, 91.05) | 3.15 (0.27, 36.48) |
| Spindle 2017 | United States | Insufficient data to calculate | 1.16 (0.11, 12.36) |
| **Outcome:** *ENNDS (non-nicotine containing e-cigarette) use at baseline and cigarette use (current or ever) at follow-up* | | | |
| Kinnunen 2019 | Finland | 1.89 (0.24, 14.58) | 0.94 (0.21, 4.12) |
| Treur 2018 | Netherlands | Insufficient data to calculate | 5.40 (2.75, 10.60) |
