# Supplementary material for: Association between electronic nicotine delivery systems and electronic non-nicotine delivery systems with initiation of tobacco use in individuals aged < 20 years. A systematic review and meta-analysis
Source: PLoS One. 2021 Sep 8;16(9):e0256044. doi: 10.1371/journal.pone.0256044 (PMC8425526; doi:10.1371/journal.pone.0256044)
Supplement: S3 Table — (DOCX) [file pone.0256044.s009.docx]

**S3 Table. Unadjusted and adjusted risk ratios for association between ENDS/ENNDS and other tobacco products**

| **Author (year)** | **Geographical area** | **Outcome** | **Unadjusted RR**  **(95% CI)** | **Adjusted RR**  **(95% CI)** |
| --- | --- | --- | --- | --- |
| Barrington-Trimis 2016 | Southern California, USA | Ever use pipes | 7.15 (1.49, 34.31) | 8.32 (1.20, 57.04) |
|  |  | Ever use cigars | 3.67 (1.76, 7.65) | 4.65 (1.77, 11.97) |
|  |  | Ever use hookah | 2.14 (1.12, 4.07) | 3.09 (1.24, 7.48) |
|  |  | Ever use any  tobacco product | 2.92 (1.85, 4.61) | 5.67 (2.61, 12.04) |
| Hansen 2020b | Germany | Ever use hookah | Insufficient data to calculate | 4.98 (3.20, 7.71) |
| Leventhal 2015 | USA | Ever use hookah | 3.43 (2.39, 4.91) | 2.36 (1.61, 3.47) |
|  |  | Ever use cigars | 5.16 (3.57, 7.44) | 3.12 (2.08, 4.65) |
|  |  | Ever use any tobacco product | 4.53 (3.37, 6.09) | 2.87 (2.08, 3.95) |
| Penzes 2018 | Romania | Waterpipe | 3.53 (2.49, 4.98) | 1.55 (1.07, 2.23) |
