# Supplementary material for: Association between electronic nicotine delivery systems and electronic non-nicotine delivery systems with initiation of tobacco use in individuals aged < 20 years. A systematic review and meta-analysis
Source: PLoS One. 2021 Sep 8;16(9):e0256044. doi: 10.1371/journal.pone.0256044 (PMC8425526; doi:10.1371/journal.pone.0256044)
Supplement: S1 Appendix — (DOCX) [file pone.0256044.s010.docx]

**S1 Appendix: Search Strategy**

**Search strategy for the review in MEDLINE**

Database(s): **Ovid MEDLINE(R) and Epub Ahead of Print, In-Process & Other Non-Indexed Citations and Daily**1946 to August 31st 2020

| **#** | **Searches** |
| --- | --- |
| 1 | Electronic Nicotine Delivery Systems/ |
| 2 | e-cig*.mp. |
| 3 | (electr* adj2 cig*).mp. |
| 4 | (electr* adj2 nicotine).mp. |
| 5 | (nicotine adj2 delivery).mp. |
| 6 | ((ENND* or END*) adj3 nicotine).mp. |
| 7 | (vape or vaping).mp. |
| 8 | e-liquid.mp. |
| 9 | e-nicotine.mp. |
| 10 | electronic hookah*.mp. |
| 11 | e-hookah*.mp. |
| 12 | e-shisha*.mp. |
| 13 | e-waterpipe*.mp. |
| 14 | 1 or 2 or 3 or 4 or 5 or 6 or 7 or 8 or 9 or 10 or 11 or 12 or 13 |
| 15 | Cross-Sectional Studies/ |
| 16 | Prevalence/ |
| 17 | (cross-sectional or prevalence or transversal).tw,kw. |
| 18 | Cohort Studies/ |
| 19 | Longitudinal Studies/ |
| 20 | Follow-Up Studies/ |
| 21 | Prospective Studies/ |
| 22 | Retrospective Studies/ |
| 23 | cohort.tw,kw. |
| 24 | longitudinal.tw,kw. |
| 25 | prospective.tw,kw. |
| 26 | retrospective.tw,kw. |
| 27 | Epidemiologic Studies/ |
| 28 | Case-Control Studies/ |
| 29 | Control Groups/ |
| 30 | Matched-Pair Analysis/ |
| 31 | ((case* adj5 control*) or (case adj3 comparison*) or control group*).tw,kw. |
| 32 | evaluation study/ |
| 33 | Evaluation Studies as Topic/ |
| 34 | Program Evaluation/ |
| 35 | Validation Studies as Topic/ |
| 36 | ((pre- adj5 post) or (pretest adj5 posttest) or (program* adj6 evaluat*)).tw,kw. |
| 37 | (effectiveness or intervention*).tw,kw. |
| 38 | tobacco survey*.mp. |
| 39 | 15 or 16 or 17 or 18 or 19 or 20 or 21 or 22 or 23 or 24 or 25 or 26 or 27 or 28 or 29 or 30 or 31 or 32 or 33 or 34 or 35 or 36 or 37 or 38 |
| 40 | adolescent/ or child/ |
| 41 | Young Adult/ |
| 42 | (child* or teen* or youth* or juvenile* or "pre adult*").mp. |
| 43 | (young adj2 (adult* or person* or individual* or people* or population* or man or men or wom?n)).mp. |
| 44 | (emerging adj2 (adult* or person*)).mp. |
| 45 | student*.mp. |
| 46 | 40 or 41 or 42 or 43 or 44 or 45 |
| 47 | 14 and 39 and 46 |
| 48 | animals/ not humans/ |
| 49 | 47 not 48 |
| **50** | **limit 49 to yr="2016 -Current"** |
